# Capsular Genotype and Lipooligosaccharide Class Associated Genomic Characterizations of *Campylobacter jejuni* Isolates From Food Animals in China

**DOI:** 10.3389/fmicb.2021.775090

**Published:** 2021-11-22

**Authors:** Xiaoqi Zang, Hongyue Lv, Haiyan Tang, Xinan Jiao, Jinlin Huang

**Affiliations:** ^1^Jiangsu Key Laboratory of Zoonosis, Jiangsu Co-innovation Center for Prevention and Control of Important Animal Infectious Diseases and Zoonoses, Yangzhou University, Yangzhou, China; ^2^Key Laboratory of Prevention and Control of Biological Hazard Factors (Animal Origin) for Agrifood Safety and Quality, Ministry of Agriculture of China, Yangzhou, China; ^3^Joint International Research Laboratory of Agriculture and Agri-Product Safety, Ministry of Education of China, Yangzhou, China

**Keywords:** *Campylobacter jejuni*, capsular genotype, lipooligosaccharide class, multilocus sequence typing, enteritis and Guillain–Barré syndrome, isolates from food animal

## Abstract

*Campylobacter jejuni* (*C. jejuni*) is the leading causative agent of gastroenteritis and Guillain–Barré syndrome (GBS). Capsular polysaccharide (CPS) and lipooligosaccharide (LOS) contribute to the susceptibility of campylobacteriosis, which have been concern the major evaluation indicators of *C. jejuni* isolates from clinical patients. As a foodborne disease, food animal plays a primary role in the infection of campylobacteriosis. To assess the pathogenic characterizations of *C. jejuni* isolates from various ecological origins, 1609 isolates sampled from 2005 to 2019 in China were analyzed using capsular genotyping. Strains from cattle and poultry were further characterized by LOS classification and multilocus sequence typing (MLST), compared with the isolates from human patients worldwide with enteritis and GBS. Results showed that the disease associated capsular genotypes and LOS classes over-represented in human isolates were also dominant in animal isolates, especially cattle isolates. Based on the same disease associated capsular genotype, more LOS class types were represented by food animal isolates than human disease isolates. Importantly, high-risk lineages CC-22, CC-464, and CC-21 were found dominated in human isolates with GBS worldwide, which were also represented in the food animal isolates with disease associated capsular types, suggesting a possibility of clonal spread of isolates across different regions and hosts. This is the first study providing genetic evidence for food animal isolates of particular capsular genotypes harbor similar pathogenic characteristics to human clinical isolates. Collective efforts for campylobacteriosis hazard control need to be focused on the zoonotic pathogenicity of animal isolates, along the food chain “from farm to table.”

## Introduction

*Campylobacter jejuni* (*C. jejuni*) is the leading cause of acute gastroenteritis in humans worldwide, posing potential risk to susceptible human and animals due to their zoonotic transmission. Watery or bloody diarrhea, abdominal pain, headache, fever, chills, and dysentery are the main symptoms of campylobacteriosis (Black et al., 1988). Gastroenteritis and Guillain–Barré syndrome (GBS) are the two representative diseases associated with human infection of *C. jejuni* (Jackson et al., 2014). Over the last 10 years, the incidence of campylobacteriosis has increased in both developed and developing countries (Ruiz-Palacios, 2007), which has generated significant threats to the public’s health. However, the precise role of *C. jejuni* in the development of clinical condition is largely unknown. Unlike other intestinal pathogens, *C. jejuni* does not harbor pathogen-defining toxins that explicitly contribute to the disease in humans. Moreover, the majority of campylobacteriosis cases are self-limiting, which makes the understanding of *Campylobacter* pathogenesis challenging (Crofts et al., 2018).

Polysaccharide capsule is an important virulence factor of clinical importance. Highly variable structures of *C. jejuni* capsular are the basis of classical Penner serotyping scheme, dividing isolates into 47 serotypes, which has been considered as the gold standard for *C. jejuni* serotyping (Penner and Hennessy, 1980). The rise of molecular diagnostic method has promoted the development capsular genotyping, re-enforcing the strong correlation between capsular polysaccharide (CPS) and Penner serotypes. This method is not sensitive to the variations in capsule gene expression or influenced by genes or gene products outside the capsule locus (Poly et al., 2011). Consequently, it has been introduced as a fast, readily available and reliable method to assess the serotypes in *C. jejuni* (Poly et al., 2015). Particular serotypes in the antecedent infection of *C. jejuni* could contribute to the development of GBS (Poly et al., 2011). HS19 (Kuroki et al., 1993), HS23/36 c (Islam et al., 2009), and HS41 (Lastovica et al., 1997; Zhang et al., 2010a) were the major serotypes commonly represented by the isolates from GBS patients in Japan, Bangladesh, South Africa, and China, respectively. HS4 (Saida et al., 1997), HS2, and HS1 were commonly prevalent in human isolates from sporadic GBS cases all over the world (Guerry et al., 2012). In contrast, HS4 complex, HS2, HS3, HS5/31, and HS8/17 were consistently prevalent in enteritis cases across all regions (Rojas et al., 2019).

Sialylated lipooligosaccharide (LOS) is another virulence factor associated with *C. jejuni* associated GBS (Godschalk et al., 2004). Molecular mimicry between sialylated *C. jejuni* LOS and the ganglioside presented on human peripheral nerve drives a cross-reactive immune response, which could result into immune-mediated nerve damage (Yuki et al., 2004). The variability of gene content in *C. jejuni* LOS biosynthesis locus has led to the assignment of several LOS locus classes (A to S) (Parker et al., 2008). Genes harbored in LOS A, B, and C classes are involved in the synthesis of sialylated LOS. In particular, LOS A class is highly dominant in GBS-associated *C. jejuni* populations, whereas LOS B, C, and E classes show over-representation in enteritis-associated *C. jejuni* populations (Ellström et al., 2016; Hameed et al., 2020).

Currently, capsular genotype and LOS class are mainly used to characterize human clinical isolates, but not animal isolates, even though campylobacteriosis is a foodborne disease in which foods of animal origin, especially poultry and cattle, play an important role (Guirado et al., 2020). In this study, a collection of animal strains sampled from a long time span and a wide range of putative hosts was identified by capsular genotyping and LOS classification, compared with the control isolates from enteritis patients. Correlation between LOS class and disease associated capsular genotype in food animal isolates were analyzed. Genetic relationship between the animal isolates with disease associated capsular genotypes and human clinical isolates worldwide were further investigated.

## Materials and Methods

### Bacterial Strains and Culture Condition

A collection of 1609 *C. jejuni* strains were isolated from the fecal samples of animals and enteritis patients in Jiangsu province in eastern China, between 2005 and 2019. Jiangsu province is a community of approximately 29,910,849 households made-up of 84,748,016 individuals. Cattle and poultry are the major food-producing species. In detail, 181 cattle isolates were sourced from two large-scale cattle farms, which were selected as the suppliers for cattle slaughterhouses. A total of 1084 poultry isolates were sampled from two large-scale poultry farms and four medium-scale poultry farms. Approximately 150–200 chicken were housed in each single pen in large-scale poultry farm, while 30–50 chicken were housed in each single pen in medium-scale poultry farm. Household rearing of pet in this area is usual, a total of 55 isolates were collected in the pets from different citizen families from 2005 to 2019. Moreover, 52 monkey isolates were sampled from the rhesus macaques (*Macaca mulatta*) at a primate neurobiology research institute from 2017 to 2018. A collection of 233 clinical isolates were sampled from the enteritis patients in three representative hospitals from 2005 to 2006, as previously reported (Huang et al., 2009). The sampling procedure was approved by the Research Ethics Committee of Yangzhou University.

*Campylobacter jejuni* isolates were routinely cultured onto *Campylobacter* selective agar base plates (modified CCDA, Preston; Oxoid, United Kingdom) under microaerophilic conditions (5% O_2_, 10% CO_2_, and 85% N_2_) at 42°C for 48 h. Isolate was identified at *C. jejuni* species level by PCR, and then stored at −80°C in brain heart infusion broth with 15% glycerol until use (Zang et al., 2017).

### Capsule Multiplex Typing Scheme

Template preparation (Huang et al., 2017) and capsule genotyping (Poly et al., 2011; Liang et al., 2016) were performed as previously described. Primers of 20 common capsular genotypes were shown in Supplementary Table 1. Five GBS associated capsular genotypes (HS19, HS41, HS23/36, HS4 c, and HS2) and enteritis associated capsular genotypes (HS2, HS4 c, HS5/31, HS8/17, and HS3) were selected by searching the keywords of ‘‘*Campylobacter*,’’ ‘‘serotype,’’ and ‘‘capsule genotype’’ in PubMed.^1^

### Determination of Lipooligosaccharide Class

A collection of *C. jejuni* isolates from China was randomly selected for LOS typing, including 157 cattle isolates, 235 poultry isolates, and 172 enteritis isolates. PCR assays targeted on LOS classes A–E were conducted as previously reported (Parker et al., 2008). The distribution of LOS classes as well as the correlation between LOS classes and eight disease associated capsular genotypes (mentioned in section “Capsule Multiplex Typing Scheme”) among isolates from poultry and cattle were compared with the corresponding data of isolates from enteritis patients.

### Multilocus Sequence Typing

A total of 97 *C. jejuni* isolates with disease associated capsule genotyes (HS19, HS4A c, HS8/17, HS2, and HS23/36) were selected to analysis genotype diversity, including 37 animal isolates and 28 enteritis isolates from China, as well as 32 GBS control isolates worldwide (China, *n* = 3; Netherlands, *n* = 13; United States, *n* = 2; Japan, *n* = 4; Africa, *n* = 3; Mexico, *n* = 2; Peru, *n* = 4; and Thailand, *n* = 1). These GBS isolates belonged to five kinds of capsule genotypes (HS41, HS19, HS41, HS2, and HS4 Ac), which were assigned to 15 different sequence types (STs) (such as ST-22, ST-362, ST-2993, and ST-19). MLST profiles of GBS isolates were downed from PubMLST.^2^

Multilocus sequence typing was conducted as previously reported (Dingle et al., 2001). STs and allele numbers were analyzed on *Campylobacter* PubMLST website. Housekeeping allelic profiles were analyzed by the goeBURST algorithm implemented in PHYLOViZ 2.0 to created minimum spanning tree (MST) and Neighbor Joining tree (Nascimento et al., 2017).

### Statistical Analyses

Statistical analyses presented in this manuscript were calculated using Chi-square test, including the proportional representations of *C. jejuni* capsular genotype and LOS class among isolates from different sources. Fisher’s exact test of SPSS Statistics 22 (SPSS Inc., Chicago, IL, United States) was used to test the significance of the experimental data. Statistical significance was set at *P* ≤ 0.05.

## Results

### Capsular Genotype Diversity Among Animal Isolates

**FIGURE 1:**
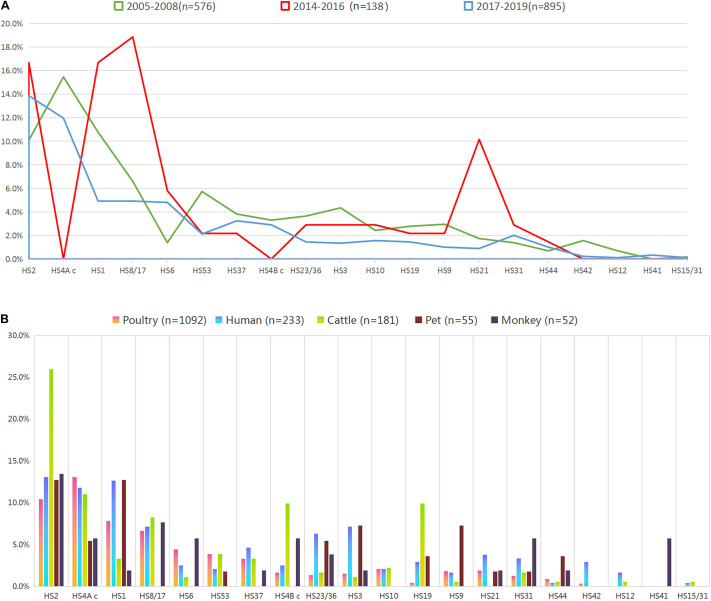
Proportional representation of capsular genotypes among *Campylobacter jejuni* isolates. **(A)** Frequency of capsular genotype among isolates from different year intervals. Colors of the lines indicate isolates from different year intervals. **(B)** Frequency of capsular genotype among isolates from enteritis patient, monkey, pet, cattle, and poultry. Colors of the bars indicate isolates from different sources.

A total of 950 isolates were serotyped, accounting for 59.04% of 1609 *C. jejuni* isolates. The rest ones included the isolates with uncommon capsular genotypes and the isolates with “non-typable (NT)” capsular genotypes (Table 1). The most four dominant capsular genotypes included HS2, HS4A c, HS1, and HS8/17, all of which reached a proportional representation of 5%. The frequency of capsular genotypes was analyzed stratifying at 3-year intervals (Figure 1A, including 2005–2008 (*n* = 576), 2014–2016 (*n* = 138), and 2017–2019 (*n* = 895). No significant difference on genotype frequency was observed among isolates from year interval 2005–2008 and 2017–2019. Notably, compared with the genotypes identified from other year intervals, six capsular genotypes were not represented by isolates from 2014 to 2016, including HS4A c, HS4B c, HS15/31, HS12, HS41, and HS42. Moreover, HS21 and HS8/17 were over-represented in isolates from 2014 to 2016, *P* < 0.05, which could be probably affected by the limitted sample size and strain source.

**TABLE 1:** Comparison of capsular genotype with proportional estimates by *Campylobacter jejuni* isolates source and collection year.

Capsular genotype	Source of *Campylobacter jejuni* isolate					Collection year			Total (*n* = 1609)
	Poultry (*n* = 1084)	Enteritis patient (*n* = 233)	Cattle (*n* = 181)	Pet (*n* = 55)	Monkey (*n* = 52)	2005–2008 (*n* = 576)	2014–2016 (*n* = 138)	2017–2019 (*n* = 895)	
HS2	10.42% (113/1084)	13.08% (31/237)	25.97% (47/181)	12.73% (7/55)	13.46% (7/52)	10.07% (58/576)	16.67% (23/138)	13.85% (124/895)	12.74% (205/1609)
HS4A c^a^	13.10% (142/1084)	11.81% (28/237)	11.05% (20/181)	5.45% (3/55)	5.77% (3/52)	15.45% (89/576)	0.00% (0/138)	11.96% (107/895)	12.18% (196/1609)
HS1	7.84% (85/1084)	12.66% (30/237)	3.31% (6/181)	12.73% (7/55)	1.92% (1/52)	10.76% (62/576)	16.67% (23/138)	4.92% (44/895)	8.02% (129/1609)
HS8/17	6.64% (72/1084)	7.17% (17/237)	8.29% (15/181)	0.00% (0/55)	7.69% (4/52)	6.60% (38/576)	18.84% (26/138)	4.92% (44/895)	6.71% (108/1609)
HS6	4.43% (48/1084)	2.53% (6/237)	1.10% (2/181)	0.00% (0/55)	5.77% (3/52)	1.39% (8/576)	5.80% (8/138)	4.80% (43/895)	3.67% (59/1609)
HS53	3.87% (42/1084)	2.11% (5/237)	3.87% (7/181)	1.82% (1/55)	0.00% (0/52)	5.73% (33/576)	2.17% (3/138)	2.12% (19/895)	3.42% (55/1609)
HS37	3.32% (36/1084)	4.64% (11/237)	3.31% (6/181)	0.00% (0/55)	1.92% (1/52)	3.82% (22/576)	2.17% (3/138)	3.24% (29/895)	3.36% (54/1609)
HS4B c^b^	1.66% (18/1084)	2.53% (6/237)	9.94% (18/181)	0.00% (0/55)	5.77% (3/52)	3.30% (19/576)	0.00% (0/138)	2.91% (26/895)	2.80% (45/1609)
HS23/36	1.38% (15/1084)	6.33% (15/237)	1.66% (3/181)	5.45% (3/55)	3.85% (2/52)	3.65% (21/576)	2.90% (4/138)	1.45% (13/895)	2.36% (38/1609)
HS3	1.57% (17/1084)	7.17% (17/237)	1.10% (2/181)	7.27% (4/55)	1.92% (1/52)	4.34% (25/576)	2.90% (4/138)	1.34% (12/895)	2.55% (41/1609)
HS10	2.12% (23/1084)	2.11% (5/237)	2.21% (4/181)	0.00% (0/55)	0.00% (0/52)	2.43% (14/576)	2.90% (4/138)	1.56% (14/895)	1.99% (32/1609)
HS19	0.46% (5/1084)	2.95% (7/237)	9.94% (18/181)	3.64% (2/55)	0.00% (0/52)	2.78% (16/576)	2.17% (3/138)	1.45% (13/895)	1.99% (32/1609)
HS9	1.85% (20/1084)	1.69% (4/237)	0.55% (1/181)	7.27% (4/55)	0.00% (0/52)	2.95% (17/576)	2.17% (3/138)	1.01% (9/895)	1.80% (29/1609)
HS21	1.94% (21/1084)	3.80% (9/237)	0.00% (0/181)	1.82% (1/55)	1.92% (1/52)	1.74% (10/576)	10.14 (14/138)	0.89% (8/895)	1.99% (32/1609)
HS5/31	1.29% (14/1084)	3.38% (8/237)	1.66% (3/181)	1.82% (1/55)	5.77% (3/52)	1.39% (8/576)	2.90% (4/138)	2.01% (18/895)	1.80% (29/1609)
HS44	0.92% (10/1084)	0.42% (1/237)	0.55% (1/181)	3.64% (2/55)	1.92% (1/52)	0.69% (4/576)	1.45% (2/138)	1.01% (9/895)	0.93% (15/1609)
HS42	0.37% (4/1084)	2.95% (7/237)	0.00% (0/181)	0.00% (0/55)	0.00% (0/52)	1.56% (9/576)	0.00% (0/138)	0.22% (2/895)	0.68% (11/1609)
HS12	0.00% (0/1084)	1.69% (4/237)	0.55% (1/181)	0.00% (0/55)	0.00% (0/52)	0.69% (4/576)	0.00% (0/138)	0.11% (1/895)	0.31% (5/1609)
HS41	0.00% (0/1084)	0.00% (0/237)	0.00% (0/181)	0.00% (0/55)	5.77% (3/52)	0.00% (0/576)	0.00% (0/138)	0.34% (3/895)	0.19% (3/1609)
HS15/31	0.00% (0/1084)	0.42% (1/237)	0.55% (1/181)	0.00% (0/55)	0.00% (0/52)	0.17% (1/576)	0.00% (0/138)	0.11% (1/895)	0.12% (2/1609)

*^*a*^HS4A complex, including HS4, HS13, and HS6.*

*^*b*^HS4B complex, including CG8486, HS16, and HS64.*

Overall, the combined proportional representation of 20 identified capsular genotypes among *C. jejuni* isolates from different sources ranged from 63.19 to 89.45% (Figure 1B). A total of 89.45% of human isolates belonged to 19 capsular genotypes, whereas 85.64% cattle isolates (155/181) belonged to 17 genotypes. Seven capsular genotypes were shared by animals isolates and human isolates, including HS2, HS4A c, HS1, HS23/36, HS3, HS31, and HS44, accounting for 40.58% of the whole isolates. Notably, particular capsular genotypes reached the representation of 5% threshold in animal isolates but lacked in human isolates, such as HS4B c and HS19 in cattle isolates, HS9 in pet isolates, HS4B c, HS31, and HS41 in monkey isolates.

### Characterization of Guillain–Barré Syndrome Associated Capsular Genotypes

**FIGURE 2:**
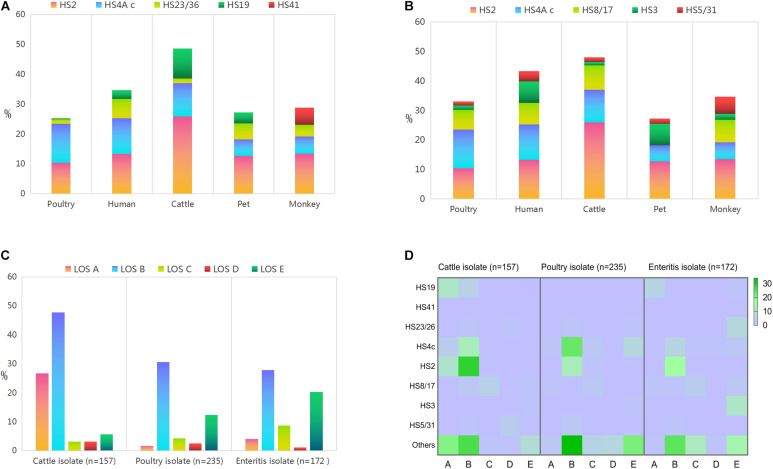
Proportional representations of the disease related capsular genotypes and lipooligosaccharide classes among *Campylobacter jejuni* isolates from different sources. **(A)** GBS associated capsular genotype. **(B)** Enteritis associated capsular genotype. **(C)** Lipooligosaccharide classes. **(D)** Heat map demonstrates the correlation between lipooligosaccharide class and disease associated capsular genotype.

Overall, GBS associated capsular genotypes accounted for 29.46% (*n* = 474) of the whole isolates (Figure 2A). The most two common genotypes were HS2 (12.74%, 205/1609) and HS4 c (12.18%, 196/1609), the frequencies of which reached a 10% threshold. In contrast, the proportional representations of the left three genotypes did not reach 3% threshold.

Notably, GBS associated capsular genotypes accounted for 48.62% (88/181) of cattle isolates, which was statistically higher than the corresponding data of isolates from other animal sources (*P* < 0.05). Frequency of HS2 was the highest in cattle isolates (25.97%, 47/181), followed by monkey isolates and human isolates. Moreover, the representation of HS4 c was highest among poultry isolates (13.10%, 142/1084), followed by human isolates. HS23/36 was dominated in human isolates (6.44%, 15/237), followed by pet isolates. HS19 was statistically dominant in isolates from cattle (9.94%, 18/181) compared to isolates from other sources (*P* < 0.05). In particular, three *C. jejuni* isolates of HS41 were only derived from monkey (100%, 3/3).

#### Characterization of Enteritis Associated Capsular Genotypes

A total of 579 isolates were characterized as enteritis associated capsular genotypes (Figure 2B), accounting for 35.99% of the whole isolates. The most three prevalent ones included HS2 (12.74%, 205/1609), HS4 c (12.18%, 196/1609), and HS8/17 (6.71%, 108/1609). The proportional representation of HS3 and HS5/31 all reached 3% threshold.

Enteritis associated capsular genotypes combined accounted for 48.07% (87/181) in cattle isolates, which was statistically higher than the corresponding data of other animal isolates (*P* < 0.05). In detail, HS8/17 reached the highest proportional representation of 8.29% (15/181) among cattle isolates, followed by monkey isolates, human enteritis isolates and poultry isolates. Moreover, HS5/31 reached the highest frequency in monkey isolates (5.77%, 3/52), whereas HS3 reached the highest proportional representation in human isolates (7.30%, 17/233).

### Lipooligosaccharide Class Diversity

Lipooligosaccharide types A–E were identified among food animal isolates (Figure 2C and Supplementary Table 2). The proportional representation of LOS class A in cattle isolates (26.75%, 42/157) was statistically higher than the corresponding data of poultry isolates (1.70%, 4/235) and human isolates (4.07%, 7/172) (*P* < 0.05). Class B was the most common LOS class locus among each source, which was mainly distributed among cattle isolates, followed by poultry isolates and human isolates. Notably, the combined frequency of LOS classes AB in cattle isolates (74.52%, 117/157) was significantly higher than poultry isolates (32.34%, 76/235) and enteritis isolates (31.98%, 55/171), *P* < 0.05.

Lipooligosaccharide class C was represented by 8.72% (15/172) human isolates, followed by cattle isolates (3.18%, 5/157) and poultry isolates (4.26%, 10/235). LOS class D did not reached a proportional representation of 3.5% among isolates from each source. In contrast, the proportional representation of LOS E class in human isolates (20.35%, 35/172) was statistically higher than the corresponding data of poultry isolates and cattle isolates (*P* < 0.05).

### Correlation Between Specific Lipooligosaccharide Classes and Disease Associated Capsular Genotype

Based on the same disease associated capsular genotype, animal isolates were distributed in more LOS types, compared with human isolates. Disease associated capsular genotypes were dominated in isolates with sialylated LOS classes ABC (Figure 2D). Among 117 cattle strains with LOS ABC classes, 61.48% isolates (*n* = 75) were identified as disease associated capsular genotypes. HS2 (31.15%, *n* = 38), HS4 c (12.30%, *n* = 15), and HS19 (11.48%, *n* = 14) were the top three prevalent capsular genotypes, followed by HS8/17, HS23/26, and HS5/31. Among the 86 poultry isolates with LOS classes ABC, the top two prevalent capsular genotypes were HS4 c (30.23%, *n* = 26) and HS2 (15.12%, *n* = 13), followed by HS8/17 (3.49%, *n* = 3), HS5/31 (2.33%, *n* = 2), and HS23/26 (1.16%, *n* = 1), the combined frequency was 52.33% (*n* = 45). In the control collection of 70 enteritis isolates with LOS ABC classes, HS2 (24.29%, *n* = 17) was the most common genotype, followed by HS4 c (8.75%, *n* = 6), HS19 (7.14%, *n* = 5), HS8/17 (7.14%, *n* = 5), and HS5/31 (1.43%, *n* = 1), the combined frequency was 48.57% (*n* = 34).

Enteritis associated capsular genotype HS3 was the most prevalent serotype among isolates with LOS E, indicating a consistency between LOS class and capsular genotype. Among the 35 enteritis isolates with LOS class E, 60% (21/35) of human isolates were identified as disease associated capsular genotypes, including HS3 (10/35, 28.57%), HS23/26 (6/35, 17.14%), HS8/17 (2/35, 5.71%), HS4c (2/35, 5.71%), and HS19 (1/35, 2.86%). In contrast, HS3 (1/9, 11.11%) and HS8/17 (1/9, 11.11%) were represented by 9 cattle isolates with LOS E class, whereas 3 capsular genotypes (HS4c, HS23/26, and HS3) were present among 29 poultry isolates with LOS E class (27.59%, *n* = 8). In this study, only three monkey isolates were identified as GBS maker serotype HS41 (mentioned in 3.2), all of which belonged to LOS class A.

### Sequence Type of *Campylobacter jejuni* With Disease Associated Capsular Genotype

**FIGURE 3:**
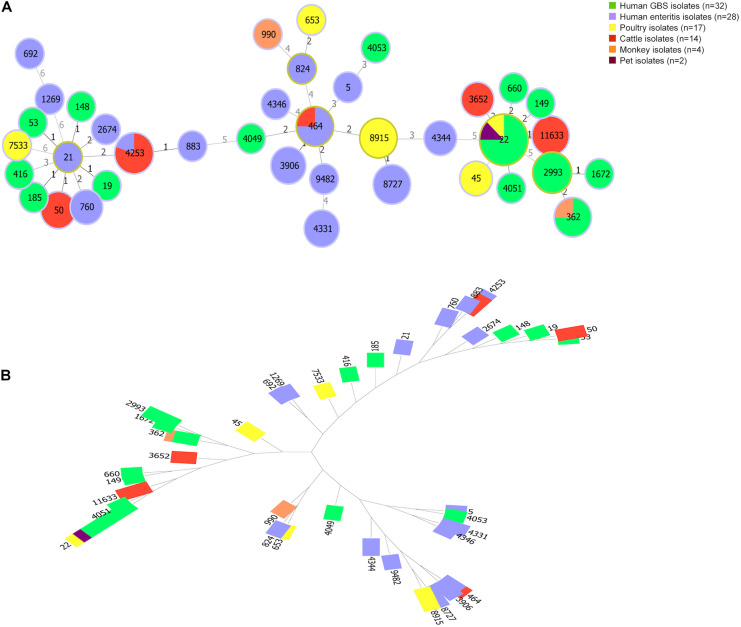
Genetic diversity of *Campylobacter jejuni* isolates from animals and patients. **(A)** The minimum spanning tree shows population structure of *C. jejuni* isolates. Circles correspond to different sequence types. The size of circle proportional to the number of isolates with a certain sequence type. Colors of the circles indicate isolates from different sources. **(B)** A tree constructed by Neighbor Joinning algorithm. Saitou–Nei criterion was selected for tree branch-length minimization.

Animal isolates (*n* = 37) were assigned to seven common clonal complexes (CCs) representing thirteen known STs. CC-22 was most dominant clonal complex (10/37, 27.03%), followed by CC-21 and CC-464. Moreover, 7 animal isolates were typed as novel STs, the MLST profiles of which did not match the known STs in MLST database (Supplementary Table 3). A comparison of MLST profiles between human isolates and animal isolates was conducted to access genetic relatedness (Figure 3). Human enteritis isolates (*n* = 28) clustered into 8 CCs represented by 16 STs and 1 novel ST. CC-464 (11/28, 39.2%) and CC-21 (6/28, 21.4%) were the most common ones. In contrast, regarding to 32 GBS isolates worldwide, 15 different STs were identified, which were classified into seven CCs, the most common one was CC-22 (15/32, 46.8%), followed by CC-362 (8/32, 25.0%) and CC-21 (4/32, 12.5%). These GBS strains of CC-22 were mainly isolated from Netherlands, followed by Japan, China, United States, and Mexico, whereas GBS isolates of CC-21 were all isolated from Netherlands. Notably, although GBS isolates were sampled all over the world, whereas animal isolates and enteritis isolates were sampled in China, zero allele distance was observed among isolates from different countries and species, using the seven house-keeping genes in MLST, such as the isolates with ST-4253 from cattle and enteritis, isolates with ST-464 from cattle and patients with enteritis, isolates with ST-22 from GBS patients, poultry, and pet, as well as isolates with ST-362 from GBS patients and monkey. Our result shown a close genetic relationship between animal isolates and human disease isolates.

**FIGURE 4:**
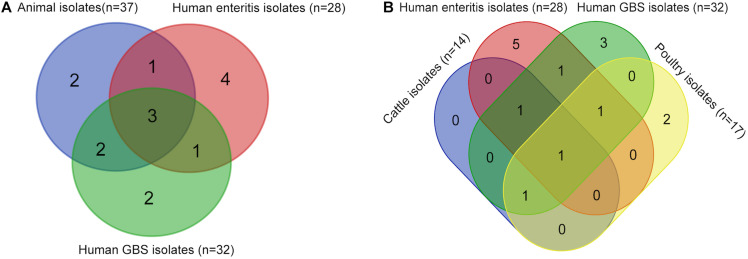
Comparison of sequence types among *Campylobacter jejuni* isolates from different sources. These isolates include 37 animal isolates with disease associated serotypes, 28 enteritis isolates, and 32 GBS isolates from PubMLST. **(A)** Venn diagram shows the sequence types of isolates from GBS patients, enteritis patients, and animals. **(B)** Venn diagram shows the sequence types of isolates from GBS patients, enteritis patients, poultry, and cattle.

Clonal complex diversities among isolates from food animal and human were showed in Venn diagram (Figure 4A and Supplementary Table 4). ST-21 complex and ST-464 complex were represented by isolates from all sources. ST-354 complex and ST-45 complex were unique for animal isolates. Moreover, four CCs (ST-1034 complex, ST-692 complex, ST-574 complex, and ST-607 complex) were only observed among enteritis isolates, whereas two CCs (ST-42 complex and ST-48 complex) were only represented by GBS isolates. In particular, all of the CCs represented by cattle isolates were also observed among GBS isolates (Figure 4B and Supplementary Table 5). ST-354 complex and ST-45 complex were unique represented by poultry isolates. Notably, a definite correlation between *C. jejuni* capsular genotype and CC distribution could be suggested by our study, strains with HS19 could be genetically related to *C. jejuni* population of ST-22 complex, whereas strains with HS41 could be genetically related to *C. jejuni* population of ST-362 complex.

## Discussion

Campylobacteriosis is a worldwide public health problem with numerous socio-economic impacts, ranging from mild symptoms to fatal illness (Hansson et al., 2018), and it is clear that not all *C. jejuni* strains are equally important as human pathogens (Nichols et al., 2012). To our knowledge, this is the first comprehensive genomic epidemiological study to reveal the genetic diversity and pathogenic correlation of *C. jejuni* isolates from various animals in China, within a long sampling time span. The characterization of the genetic pathogenicity of *C. jejuni* is essential for better infection control practice and clinical treatment in humans. Here, we observed that part of animal isolates shared disease associated capsular genotypes with human clinical isolates. Notably, these zoonotic isolates also belonged to the dominant CCs which were over-represented in the clinical isolates from GBS and enteritis patients, specifically CC-22 and CC-21. Notably, CC-21 is among the most common causes of acute human infection (Sheppard et al., 2009), while CC-22 isolates account for up to a third of infections among patients who developed GBS following campylobacteriosis (Islam et al., 2009). Notably, ST22 clonal complex has also been described as “high-risk” lineage, which was also over-represented in isolates that lead to the development of *Campylobacter* enterocolitis associated post-infection-Irritable Bowel Syndrome in United States (Peters et al., 2021). In this study, the same “high-risk” lineage was also observed among the animal isolates from China and the GBS isolates worldwide (Netherlands, United States, Mexico, Japan, and China), indicating the pathogenic potential of zoonotic isolates need to be highlighted.

Our current data revealed that disease associated serotype reached the highest proportional representation in *C. jejuni* isolates from cattle, which enhanced our understanding of the pathogenic potential from zoonotic isolates. Recently, human isolates have been reported being closely related to cattle isolates, although the epidemiological meta data necessary to determine causality were unavailable (Hsu et al., 2020). Based on the idea of “one health” (Wolfe et al., 2007), human infection of zoonotic disease needs to be controlled from animals. The source of human infection is thought to be the massive reservoir of *C. jejuni* in animal population (Parker et al., 2005), since *C. jejuni* isolates colonize in the intestines of various hosts. While exposure to retail chicken meat has been reported as the major risk factor of campylobacteriosis (Huang et al., 2017), many isolates from poultry carcasses and products did not show genetic relatedness to human pathogenic strains (Zhang et al., 2010b). Notably, drinking of unpasteurized milk and contact with domestic animals could be the other ways of *C. jejuni* propagation (Wilson et al., 2008). Our results showed that the frequency of disease associated serotypes combined was statistically higher in cattle isolates compared to isolates of other animals. HS19 was especially over-represented in cattle isolates, which has been reported as the major serotype prevalent in *C. jejuni* isolates from GBS patients (Heikema et al., 2015).

Capsular genotype and LOS class have been concerned the two direct indicators of campylobacteriosis. Association between LOS class and disease associated capsular genotype was of considerable interest for a better understanding of *C. jejuni* pathogenesis mechanism. Striking genetic correlation has been found between human clinical isolates with GBS associated serotypes and LOS classes (Penner et al., 1983). In our research, this correlation was not only confirmed by human clinical isolates but also observed among animal isolates. Specially, HS19 was prevalent in the enteritis isolates with LOS classes AE, which was also over-represented in cattle isolates with LOS AB classes. In contrast, HS23/26 was dominant in enteritis isolates with LOS E class, which was also present in cattle and poultry isolates with LOS B class. Moreover, GBS maker serotype HS41 was only found in the monkey isolates belonging to the LOS A class, indicating a potential hazard of animal origin.

Although no evident genetic linkage between LOS classification and capsular genotyping in enteritis cases has been previously reported (Karlyshev et al., 2005), our results showed that LOS E was the second dominated LOS class in enteritis isolates, followed by LOS B. Enteritis associated serotype HS3 was solely associated with enteritis isolates of LOS E class, indicating a correlation attributed to the genes shared by LOS and capsules. The bi-functionality of some enzymes involved in polysaccharide biosynthesis may additionally explain why particular LOS and capsule genotypes are linked, associated genes included *cj1152* and *gmhA2* (Karlyshev et al., 2005). More in-depth studies on gene functionalities of the biosynthesis genes of LOS and capsule are needed to explain why certain LOS classes and capsules are genetically linked together (Heikema et al., 2015). Moreover, our results showed that, unlike the sialylation of isolates with LOS class A could trigger GBS, LOS class E is not characterized as sialylation, indicating sialylation is not required for human diarrheal disease, which was is consistent with the previously reported one (Hameed et al., 2020), both the sialylated and non-sialylated LOS can be used for vaccine design.

Capsule multiplex PCR approach has been introduced as a fast, readily available and reliable method, allowing high-throughput genotyping of a large dataset of isolates within several hours (Poly et al., 2015). In contrast, whole genome sequencing (WGS) has emerged as an effective method to examine the genomic characteristic of *Campylobacter* isolates with high-resolution, novel types of “non-typable” isolates could be possibly identified. Multiplex PCR approach and WGS analysis offer complementary strengths for isolates identification. WGS could provide isolates with huge genomic information, at present, it takes at least 1 month for isolates sequencing, including the preparation of bacterial DNA, establishment of DNA library, sequencing, reads assembly, and genome annotation. In this study, since we mainly focused on the prevalence of disease associated genotypes among a large dataset of *C. jejuni* isolates, we chose PCR approach for serotype characterization. Besides of, the serotyped isolates, the left isolates could belong to the uncommon serotypes exist in Penner serotyping scheme but not be involved in this study, as well as the undiscovered serotypes. In fact, non-typable isolates have been consistently reported since Penner first introduced serotyping scheme. The phase variable nature of CPS expression in *C. jejuni*, especially the exchange of capsular genes by horizontal gene transfer could generate new capsular genotypes (Bacon et al., 2001; Karlyshev et al., 2005; Clarke et al., 2021). In the future, isolates with undiscovered genotypes will be sequenced, CPS gene clusters of these isolates will be investigated using comparative genomics techniques. Paralog or unique sequences in CPS gene cluster could be extracted and be employed as the amplification target for capsular genotypes identification.

Epidemiological data could provide accurate assessment of the burden of campylobacteriosis. In this study, for a better understanding of the pathogenic potential of food animal isolates, *C. jejuni* isolates collected from representative putative animal hosts within a long sampling time span were genotyped. One drawback of this study is that the quantity of human isolates was much smaller than animal isolates. Largely due to most of the campylobacteriosis cases are self-limiting as well as the rigorous cultivation condition of *C. jejuni*, disease associated human strain has been still insufficient in Africa and Asia, not to mention about the prevalence information of capsular types (Islam et al., 2009). In view of the quantity of human isolates, the reported human disease associated capsule and lipooligosaccharide types were especially identified, domestic and foreign human isolates from enteritis and GBS patients were involved in this study as the control isolates. As *C. jejuni* associated food safety problems have been increasingly highlighted, more and more human isolates will be monitored and characterized through the collaboration between hospitals and research institutes. More human isolates will be identified in our future research to confirm or disprove the assumption generated from this study. Moreover, animal isolates from a wider geographically area (other regions in China and other countries) will also be characterized using genotyping and *in vitro* pathogenic experiment.

Campylobacteriosis is a mainly foodborne disease, food animal plays a primary role. Firstly, this study contributed to a better understanding of the pathogenicity potential of representative food animal isolates, which were collected from a long sampling time span and a wide range of putative hosts. Secondly, distributions of disease associated capsular genotypes and LOS classes in animal isolates were firstly studied. Correlations between LOS class, capsular genotypes and CCs were investigated. High-risk lineages were found dominated in the isolates with disease associated capsular types, including the isolates from GBS patients worldwide and food animals in China, suggesting the possibility of clonal spread of the disease associated capsular genotype isolates across different regions and hosts. Last but not least, our results not only confirmed the previously reported genetic relatedness between cattle isolates and human pathogenic strains, but also indicated that disease associated capsular genotypes and LOS classes all reached a higher frequency in cattle isolates than poultry isolates, providing genetic evidence for these food animal isolates harbor human clinical isolates alike pathogenic characteristics. Generally speaking, this study provided critical supporting data to understand the hazard of *C. jejuni* isolates from food animals, suggesting cattle isolate with disease associated capsular genotypes is especially need to be eliminated for food safety control, which will lay a foundation for the development of campylobacteriosis biocontrol in animal sector.

## Data Availability Statement

All data needed to evaluate the conclusions of this article are present in the article. MLST profiles of 65 isolates in this study have been uploaded in PubMLST2, with new ID: 110484-110548. Five new sequence types include ST-11157, ST-11159, ST-11160, ST-11161, and ST-11162.

## Ethics Statement

This study was carried out in accordance with the principles of the Basel Declaration and recommendations of the institutional administrative committee and ethics committee of laboratory animals. License was issued by the Science and Technology Department of Jiangsu Province in China [SYXK (Su) 2017-0044]. The sampling procedure was supervised and inspected by the Animal Welfare and Ethics Committee of Yangzhou University, with the guidelines of the National Institutes of Health Guide for the Care and Use of Laboratory Animals (NIH publication number 80–23).

## Author Contributions

XZ: conceptualization, writing – original draft preparation, writing – review and editing, and visualization. XZ and HT: methodology and formal analysis. XZ and HL: investigation. JH and XJ: project administration. JH: funding acquisition. All authors have read and agreed to the published version of the manuscript.

## Conflict of Interest

The authors declare that the research was conducted in the absence of any commercial or financial relationships that could be construed as a potential conflict of interest.

## Publisher’s Note

All claims expressed in this article are solely those of the authors and do not necessarily represent those of their affiliated organizations, or those of the publisher, the editors and the reviewers. Any product that may be evaluated in this article, or claim that may be made by its manufacturer, is not guaranteed or endorsed by the publisher.

## Footnotes

1
https://pubmed.ncbi.nlm.nih.gov/?

2
http://pubmlst.org/campylobacter
