# Supplementary material for: Capsular Genotype and Lipooligosaccharide Class Associated Genomic Characterizations of Campylobacter jejuni Isolates From Food Animals in China
Source: Front Microbiol. 2021 Nov 22;12:775090. doi: 10.3389/fmicb.2021.775090 (PMC8690235; doi:10.3389/fmicb.2021.775090)
Supplement: Supplementary file 1 [file Data_Sheet_1.PDF]

Supplementary Table 1. Summary of the primer sequences in *Campylobacter jejuni* capsule multiplex typing scheme.

Primer	Capsular genotype	Product size (bp)	Forward sequence (5'-3')	Reverse sequence (5'-3')	Targeted gene	Reference
Set A						Hao et al., 2016
HS2	HS2	780	CCCCGCCAGTAGTTAAGGT	AATGAGGCTACGATTCCGC	*cj1431c*	
HS19	HS19	649	GAATGCGTTATGAGCAACAGGAT	GATCATCATCAAGCCTTTGC	*BJ-CJGB96G25-GL000630*	
HS41	HS41	328	AGATGTATGGAAGGTATGTGGTC	TAAATGGGGTGCTCGTGAA	*ICDCCJ07001_1363*	
Set B						Hao et al., 2016
HS9	HS9	460	TCCCCATCACCATAGGCTA	ACAATCCGTCTTTCGCAAT	*BJD63GL000835*	
HS12	HS12	835	GCAGCCAAACTATAACGCATTA	AAAGTGGGCGGCAAATAGTAGA	*BJD70GL000816*	
HS21	HS21	989	TACTCCACGATACCGCAGG	TTATGGTTCTGCTTGGGCT	*1-1GL001635;1-1GL001636*	
HS37	HS37	550	GATAAGGAAAACGGCGGTCT	CAAAATGGCAATCTTCAGCA	*HB-CJGB-LLGL000322*	
Set Alpha						Fre´de´ric Poly et al. 2011
HS4A c	HS4&HS13&HS64	370	TATATTTGGTTAGGGATCCA	CCTAACATATCATACACTACGGT	*HS4.07*	
HS3	HS3	149	GGTAAGGTTGATTCTGGGTTTAAT	AGATTAGGCCAAGCAATGATAA	*HS3.17*	
HS6	HS6&HS7	185	CATACATTTGCTTTCAGATTCTTTAC	ACACGCCTATTGTTGTTGTTC	*C8J-1331*	

HS10	HS10	229	TCTTATGCAG CACGCTGAT	CAAATTCAAT CGACTAGCC ACT	*HS10.08*	
HS15	HS15&HS31	325	ACAGGTAATA AAATGTGCGA GTTT	ATGCATCTGC AACATCATCC	*HS15.12*	
HS53	HS53	251	AGGCAAGCAG GAATTGTTT	TTAATTGCTC TTTGGCAATC TT	*CJE1602*	
Set Beta						Poly et al. 2011
HS4B	CG8486, HS16&HS64	652	GTGGACATGG AACTGGGACT	AAAACGTTTA AAGTCAGTG GAAA	*CJ8486-1475*	
HS8/17	HS8&HS17	342	TTCACGTGGA GGATTATTGG	TTGAACATTT CATGTGTATT CCCTA	*HS17.16*	
HS1	HS1 c	607	TTGGCGGTAA GTTTTTGAAG A	GCAAGAGAA ACATCTCGCC TA	*HS1.08*	
HS42	HS42	441	ATGGTAAAAC CGGCATTTCA	ATGCTTCAGT TCCACCCAAA	*HS42.14*	
HS44	HS44	148	AGAAGATGCA CTAGGCTCTA G	GCTATCTAAT TCCATCCCTG	*CF496678*	
HS23/36	HS23&HS36	161	GCTTGGGAGA TGAATTTACC TTTA	GCTTTATATC TATCCAGTCC ATTATCA	*CJJ81176-1435*	

References

Liang, H., Zhang, A., Gu, Y., You, Y., Zhang, J., Zhang, M. (2016). Genetic Characteristics and Multiple-PCR Development for Capsular Identification of Specific Serotypes of *Campylobacter jejuni*. *PLoS One*. 11(10), e0165159. doi: 10.1371/journal.pone.0165159.

Poly, F., Serichatalergs, O., Schulman, M., Ju, J., Cates, C. N., Kanipes, M., et al. (2011). Discrimination of major capsular types of *Campylobacter jejuni* by multiplex PCR. *J. Clin. Microbiol.* 49(5), 1750-1757. doi: 10.1128/JCM.02348-10.

Supplementary Table 2. Distribution of Lipooligosaccharide classes of *Campylobacter jejuni* isolates.

Strain	Capsular genotype	Lipooligosaccharide class				
		A	B	C	D	E
Poultry isolates (n=235)	HS19	0	0	0	0	0
	HS41	0	0	0	0	0
	HS23/26	0	1	0	0	1
	HS4c	1	23	2	0	6
	HS2	0	12	1	0	0
	HS8/17	1	0	2	0	0
	HS3	0	0	0	0	1
	HS5/31	0	2	0	0	0
	Others[1]	2	34	5	6	21
	SUM-poultry isolates	4	72	10	6	29
Cattle isolates (n=157)	HS19	10	4	0	0	0
	HS41	0	0	0	0	0
	HS23/26	0	1	0	1	0
	HS4c	3	12	0	0	0
	HS2	9	29	0	0	0
	HS8/17	0	2	4	0	1
	HS3	0	0	0	0	1
	HS5/31	0	1	0	3	0
	Others[1]	20	26	1	1	7
	SUM-cattle isolates	42	75	5	5	9
Human isolates (n=172)	HS19	5	0	0	0	1
	HS41	0	0	0	0	0
	HS23/26	0	0	0	0	6
	HS4c	0	5	1	1	2
	HS2	0	17	0	0	0
	HS8/17	1	1	3	0	2
	HS3	0	0	0	0	10
	HS5/31	0	1	0	0	0
	Others[1]	1	24	11	1	14
	SUM-human isolates	7	48	15	2	35

[1]Others: Isolates characterized as other capsular genotypes.

Supplementary Table 3. Information of *Campylobacter jejuni* isolates for MLST analysis.

Strain	Capsular genotype	Sequence type	Location	Collection	Clonal complex
P073A	HS4A c	5	China	Human enteritis	ST-353 complex
GB14	HS2	19	Netherlands	Human GBS	ST-21 complex
P051C	HS2	21	China	Human enteritis	ST-21 complex
GB18	HS19	22	Netherlands	Human GBS	ST-22 complex
GB3	HS19	22	Netherlands	Human GBS	ST-22 complex
CjGB03	HS19	22	Netherlands	Human GBS	ST-22 complex
CjGB18	HS19	22	Netherlands	Human GBS	ST-22 complex
CjGB60	HS19	22	Netherlands	Human GBS	ST-22 complex
CjRM1245	HS19	22	USA	Human GBS	ST-22 complex
CjRM1477	HS19	22	USA	Human GBS	ST-22 complex
CjRM3147	HS19	22	Mexico	Human GBS	ST-22 complex
CjRM1510	HS19	22	Japan	Human GBS	ST-22 complex
C15-56	HS19	22	Japan	Human GBS	ST-22 complex
HB93-13	HS19	22	China	Human GBS	ST-22 complex
BJ-CJGB96G25	HS19	22	China	Human GBS	ST-22 complex
AC34	HS19	22	China	Poultry	ST-22 complex
AD18	HS19	22	China	Poultry	ST-22 complex
ADO33	HS19	22	China	Pets	ST-22 complex
AP12	HS19	22	China	Pets	ST-22 complex
HG19D	HS4A c	45	China	Poultry	ST-45 complex

HG19D	HS4A c	45	China	Poultry	ST-45 complex
B-YZU0713	HS8/17	50	China	Cattle	ST-21 complex
B-YZU0715	HS8/17	50	China	Cattle	ST-21 complex
B-YZU0721	HS8/17	50	China	Cattle	ST-21 complex
GB13	HS2	53	Netherlands	Human GBS	ST-21 complex
GB25	HS2	131	Netherlands	Human GBS	-[1]
C-YZU0749	HS2	131	China	Poultry	-
C-YZU0751	HS2	131	China	Poultry	-
GB11	HS2	148	Netherlands	Human GBS	ST-21 complex
GB28	UT[2]	149	Netherlands	Human GBS	ST-22 complex
GB5	HS4A c	185	Netherlands	Human GBS	ST-21 complex
260.94	HS41	362	Africa	Human GBS	ST-362 complex
233.94	HS41	362	Africa	Human GBS	ST-362 complex
308.95	HS41	362	Africa	Human GBS	ST-362 complex
AM19	HS41	362	China	Monkey	ST-362 complex
GB17	HS4A c	416	Netherlands	Human GBS	ST-48 complex
CN32A	HS2	464	China	Cattle	ST-464 complex
PO18D	HS2	464	China	Human enteritis	ST-464 complex
P156A	HS2	464	China	Human enteritis	ST-464 complex
P18E	HS2	464	China	Human enteritis	ST-464 complex
CH02B	HS2	653	China	Poultry	ST-354 complex
CjGB28	HS19	660	Netherlands	Human GBS	ST-22 complex
P224D	HS2	692	China	Human enteritis	ST-692 complex

P116B	HS8/17	760	China	Human enteritis	ST-21 complex
P116B	HS8/17	760	China	Human enteritis	ST-21 complex
P83E	HS2	824	China	Human enteritis	ST-257 complex
P12E	HS2	883	China	Human enteritis	ST-21 complex
M155C	HS23/36	990	China	Monkey	ST-257 complex
P442C	HS2	1269	China	Human enteritis	ST-1034 complex
RM3148	HS41	1672	Mexico	Human GBS	ST-42 complex
P-267B	HS8/17	2674	China	Human enteritis	ST-21 complex
11853	HS41	2993	Peru	Human GBS	ST-362 complex
11854	HS41	2993	Peru	Human GBS	ST-362 complex
11855	HS41	2993	Peru	Human GBS	ST-362 complex
11856	HS41	2993	Peru	Human GBS	ST-362 complex
ICDCCJ07001	HS41	2993	China	Human GBS	ST-362 complex
AB22	HS19	3652	China	Cattle	ST-22 complex
AB25	HS19	3652	China	Cattle	ST-22 complex
PS3E	HS2	3906	China	Human enteritis	ST-464 complex
P161C	HS2	3906	China	Human enteritis	ST-464 complex
P16E	HS2	3906	China	Human enteritis	ST-464 complex
KB2749	HS19	4049	Japan	Human GBS	ST-464 complex
KB3445	HS19	4051	Japan	Human GBS	ST-22 complex
CJ-1667	HS19	4053	Thailand	Human GBS	ST-353 complex
C154A	HS2	4253	China	Cattle	ST-21 complex

PN42A	HS2	4253	China	Cattle	ST-21 complex
B-YZU0725	HS2	4253	China	Cattle	ST-21 complex
B-YZU0790	HS2	4253	China	Cattle	ST-21 complex
PN42A	HS2	4253	China	Human enteritis	ST-21 complex
P181D	HS2	4331	China	Human enteritis	ST-607 complex
P348D	HS2	4331	China	Human enteritis	ST-607 complex
P38E	HS2	4331	China	Human enteritis	ST-607 complex
P199D	HS4A c	4344	China	Human enteritis	ST-574 complex
P199D	HS4A c	4344	China	Human enteritis	ST-574 complex
P611D	HS8/17	4346	China	Human enteritis	-
AC24	HS19	7533	China	Poultry	-
PP69A	HS2	8727	China	Human enteritis	ST-464 complex
B-YZU0729	HS2	8727	China	Human enteritis	ST-464 complex
P16D	HS2	8727	China	Human enteritis	ST-464 complex
P501D	HS2	8727	China	Human enteritis	ST-464 complex
CH15B	HS2	8915	China	Poultry	ST-464 complex
CH17B	HS2	8915	China	Poultry	ST-464 complex
CH13B	HS2	8915	China	Poultry	ST-464 complex
CH19B	HS2	8915	China	Poultry	ST-464 complex
P157A	HS2	9482	China	Human enteritis	ST-464 complex
BB09	HS19	11633	China	Cattle	ST-22 complex
BB14	HS19	11633	China	Cattle	ST-22 complex
BB15	HS19	11633	China	Cattle	ST-22 complex

BB16	HS19	11633	China	Cattle	ST-22 complex
C-G017A	HS2	UT[2]	China	Poultry	-
M-YZU1628	HS2	UT	China	Monkey	-
M-YZU1629	HS2	UT	China	Monkey	-
G017A	HS2	UT	China	Poultry	-
CH10A	HS2	UT	China	Poultry	-
P913B	HS23/36	UT	China	Human enteritis	-
BD21	HS19	UT	China	Poultry	-
AG29	HS19	UT	China	Poultry	-

[1]-: Unknown clonal complex.
[2]UT: Novel sequence type, which that did not match known sequence type in the MLST database.

Supplementary Table 4. Sequence type complex of *Campylobacter jejuni* isolates from GBS patients, enteritis patients, and animals.

Collections	Total number	Sequence type complex
Animal isolates(n=37);<br>Human GBS (n=32);<br>Human enteritis (n=28)	3	-[1]<br>ST-21 complex<br>ST-464 complex
Animal isolates(n=37);<br>Human enteritis (n=28)	1	ST-257 complex
Animal isolates(n=37);<br>Human GBS (n=32)	2	ST-22 complex<br>ST-362 complex
Human GBS (n=32);<br>Human enteritis (n=28)	1	ST-353 complex
Animal isolates(n=37)	2	ST-354 complex<br>ST-45 complex
Human enteritis (n=28)	4	ST-1034 complex<br>ST-692 complex<br>ST-574 complex<br>ST-607 complex
Human GBS (n=32)	2	ST-42 complex<br>ST-48 complex

[1]-: Unknown sequence type complex.

Supplementary Table 5. Sequence type complex of *Campylobacter jejuni* isolates from GBS patients, enteritis patients, poultry and cattle.

Collections	Total number	Sequence type complex
Human GBS (n=32);<br>Human enteritis (n=28);<br>Poultry (n=17);<br>cattle isolates(n=14)	1	ST-464 complex
Human GBS (n=32);<br>Human enteritis (n=28);<br>cattle isolates(n=14)	1	ST-21 complex
Human GBS (n=32);<br>Poultry (n=17);<br>cattle isolates(n=14)	1	ST-22 complex
Human GBS (n=32);<br>Human enteritis (n=28);<br>Poultry (n=17)	1	-[1]
Human GBS (n=32);<br>Human enteritis (n=28);	1	ST-353 complex
Human enteritis (n=28)	5	ST-1034 complex<br>ST-692 complex<br>ST-574 complex<br>ST-257 complex<br>ST-607 complex
Human GBS (n=32)	3	ST-42 complex<br>ST-48 complex<br>ST-362 complex
Poultry (n=17)	2	ST-354 complex<br>ST-45 complex

[1]-: Unknown sequence type complex.
